# Predictors of Postoperative Hospital-Acquired Infection and Mortality Following Cardiac Surgery in a Low-Income Country: A Retrospective Cohort Study

**DOI:** 10.21470/1678-9741-2024-0111

**Published:** 2025-03-17

**Authors:** Camila Sales Fagundes, Diego Chemello, Luana Quintana Marchesan, Vitória Carolina Kohlrausch, Rafael Fortes Locateli, Eduardo Porto Santos, Isabella Klafke Brixner, Valéria Maria Limberger Bayer, Mateus Diniz Marques

**Affiliations:** 1 Department of Clinical Medicine, Universidade Federal de Santa Maria, Santa Maria, Rio Grande do Sul, Brazil

**Keywords:** Cardiac Surgical Procedures, Hospitalization Infection, Mortality

## Abstract

**Introduction:**

Recognizing the risk factors for postoperative hospital-acquired infection and mortality is crucial for better outcomes. We aimed to determine the risk predictors for postoperative hospital-acquired infection and death following cardiac surgery.

**Methods:**

This is a retrospective cohort study that included 880 consecutive adult patients who underwent cardiac surgery between 2015 and 2021. Multivariable logistic regression was performed to assess the predictors of postoperative hospital-acquired infection and mortality.

**Results:**

Patients who developed postoperative hospital-acquired infection had higher values on European System for Cardiac Operative Risk Evaluation score (4.01% vs. 2.51%; P=0.001), as well as longer hospital preoperative stay (9.44 vs. 8.28 days; P=0.049) and hospital length of stay (28.41 vs. 16.16 days; P<0.001). After multivariable analysis, predictors of postoperative hospital-acquired infection were longer hospital preoperative stay (odds ratio 1.024; 95% confidence interval 1.005-1.044; P=0.009), higher body mass index (odds ratio 1.043; 95% confidence interval 1.008-1.079; P=0.015), and longer extracorporeal circulation time (odds ratio 1.007; 95% confidence interval 1.003-1.012; P<0.001). Both longer extracorporeal circulations time and postoperative hospital-acquired infection were significantly associated with higher mortality before hospital discharge (odds ratio 1.012; 95% confidence interval 1.006-1.019; P<0.001; and odds ratio 2.418; 95% confidence interval 1.385-4.233; P=0.001, respectively).

**Conclusion:**

Extended preoperative hospitalization, body mass index, and extracorporeal circulation time are correlated with heightened postoperative hospital-acquired infection rates. Moreover, longer extracorporeal circulation time and postoperative hospital-acquired infection incidence emerged as significant predictors of mortality following cardiac surgery.

## INTRODUCTION

**Table:** 

Abbreviations, Acronyms & Symbols				
BMI	= Body mass index		INR	= International normalized ratio
CABG	= Coronary artery bypass grafting		NSTEMI	= Non-ST-elevation myocardial infarction
CHF	= Congestive heart failure		OR	= Odds ratio
CI	= Confidence interval		PAD	= Peripheral arterial disease
COPD	= Chronic obstructive pulmonary disease		PHAI	= Postoperative hospital-acquired infection
CPB	= Cardiopulmonary bypass		SAH	= Systemic arterial hypertension
DM	= Diabetes mellitus		SSI	= Surgical site infections
ECC	= Extracorporeal circulation		STEMI	=ST-elevation myocardial infarction
EuroSCORE	= European System for Cardiac Operative Risk Evaluation		TIA	= Transient ischemic attack
HUSM	= Hospital Universitário de Santa Maria		VR	= Valve replacement
ICU	= Intensive care unit			

Infections following cardiac surgery represent a significant concern within the medical community, given their potential to undermine patient outcomes and impose substantial burdens on healthcare resources^[1]^. A primary concern associated with infections after cardiac surgery is the increased morbidity and mortality rates observed among affected individuals^[2-4]^. Postoperative hospital-acquired infections (PHAI) have been documented from 5 to 20% after cardiac surgery^[4]^. The most important infection sites reported are ventilator-associated pneumonia at 5.5 to 8%, urinary tract infections at 3 to 8%, surgical wound infections at 0.5 to 8%, and sepsis at 3 to 5%^[4,5]^.

Most studies in this field have relied on small sample sizes and have emerged from developed countries, notably the United States of America and Europe^[6]^. Patients who contract PHAI tend to experience heightened hospitalization costs - often driven by the need for extended medical care -, specialized treatments, and additional resources^[5,7]^. These escalated costs not only impact on the financial well-being of patients but also contribute to the broader financial burden borne by healthcare systems^[5]^. Information regarding the economic consequences of infections in developing countries remains limited^[6]^. Evaluating these dimensions, particularly within public hospitals associated with the Brazilian public health system, can provide substantial contributions toward the distribution of financial resources^[1]^.

Therefore, we present a retrospective analysis regarding the incidence of infections and mortality after cardiac surgery in a tertiary public Brazilian hospital. We evaluate predictors of PHAI and mortality following cardiac surgery. This study approaches the healthcare-system experience in a developing country, which represents a significant portion of the world's population.

## METHODS

### Study Design

This study included consecutive adult patients ( ≥ 18 years old) who underwent cardiac surgery procedure requiring extracorporeal circulation (ECC) at the Hospital Universitário de Santa Maria (HUSM), Brazil, between July, 2015 and April, 2021. The procedures included coronary artery bypass grafting (CABG), valve replacement, ascending aorta replacement, combined CABG and valve replacement, ascending aorta repair or atrial septal defect repair. Situated in the southern region of Brazil, the HUSM serves as a referral center for approximately 1.2 million people^[8]^. The exclusion criteria for the analysis of PHAI included patients with active infections, those on therapeutic antibiotics during the procedure, and patients who died in the surgery. Prophylactic antibiotics, administered according to guidelines, were not considered exclusion criteria.

### Data Collection

Clinical information was acquired through the hospital's electronic medical records management system. The dataset included a range of preoperative variables, such as demographic details (age, sex, height, weight, body mass index [BMI], smoking history), comorbidities (diabetes mellitus [DM], chronic obstructive pulmonary disease [COPD], cerebrovascular disease, peripheral vascular disease, renal disease, atrial fibrillation, and general and cardiac surgical history), cardiac structure and function (New York Heart Association class, left ventricular ejection fraction), and laboratory measurements.

For intraoperative factors, we considered the type of surgery, blood transfusion needs, cardiopulmonary bypass (CPB) time, and ECC time. Postoperative variables encompassed the duration of mechanical ventilation, days spent in the intensive care unit (ICU) and hospital, tracheostomy, and instances of reintubation.

### Definition

Surgical site infections (SSI) cover a wide range of conditions, from surface-level scar infections to severe issues like sternal osteitis, mediastinitis, and endocarditis. At the hospital chosen for this study, the definition of SSI aligns with the guidelines set forth by the National Technical Committee on Nosocomial Infections and Care-Related Infections^[9]^. We considered patients with positive bacterial cultures following surgery as an indicator of PHAI. Medical records were used to establish SSI. At our institution, we administer Cefazolin 48 hours before cardiac surgery for prophylaxis, and this measure is not considered an indication of PHAI.

Smoking history was defined as current or prior daily smoking (with a cessation period of at least six months). Fasting blood sugar test ≥ 126 mg/dL, random glucose ≥ 200 mg/dl, or documented medical history of DM were considered to define the presence of DM. A creatinine clearance < 75 ml/min/1.73 m^2^ in men and 85 ml/min/1.73 m^2^ in women was defined as renal insufficiency.

### Operative Procedure

In the university hospital, surgical procedures adhere to established techniques outlined in the literature. Patients who underwent CABG were submitted to a traditional revascularization approach with medial sternotomy, cardioplegic arrest, and ECC support under hypothermia and standardized anticoagulation. After cross-clamp application and inducement of cardioplegia, the anastomoses were performed, including at least one internal mammary artery when feasible, with the objective of complete revascularization. Once proximal and distal anastomoses were completed, the aorta and grafts were de-aired with subsequent removal of the aortic clamp, following myocardial reperfusion. Intraoperative inotropic agents were used as necessary, as well as mechanical circulatory support. Due to the limited resources and technical considerations, intraoperative transesophageal echocardiography and off-pump procedures were not performed. For valvular surgeries, the use of mechanical or biological prostheses was left to the discretion and judgment of the medical team.

### Statistical Methods

Statistical analysis was performed using the IBM Corp. Released 2016, IBM SPSS Statistics for Windows, version 24.0, Armonk, NY: IBM Corp. The Shapiro-Wilk test was used for the determination of quantitative data distribution. Continuous variables with normal distribution were described as mean ± standard deviation. Continuous variables with non-symmetrical distribution were described as median and interquartile ranges. For the independent samples, the Student’s t-test and the nonparametric Mann-Whitney test were used for continuous variables. Categorical variables were expressed as percentages and compared by Chi-square test or Fisher's exact test. Binary logistic regression analysis was employed to identify the predictors of the outcomes. The selection of variables was based on previously reported predictive models in the literature. Significant potential predictors at the 0.1 level in the univariate analysis were included in the multivariable model, and the results were expressed as odds ratios (OR) with 95% confidence intervals (CI). To ensure the validity of the multivariable logistic regression analysis, multicollinearity was diagnosed. All statistical tests were two-tailed, and a final P-value < 0.05 was considered statistically significant.

In the univariate analysis, the sample was divided considering two outcomes: the development of PHAI (no infection and infection) and mortality before hospital discharge (no death and death). The sample for the analysis of PHAI comprised 869 patients because 11 patients were excluded due to death during surgery, rendering the analysis of PHAI infeasible for those patients. The univariate analysis related to mortality comprised all 880 patients. The binary logistic regression model was performed first considering PHAI as the grouping variable, along with the length of preoperative hospital stay, BMI, and ECC time. Secondly, another binary logistic regression model was performed, considering death before hospital discharge as the grouping variable, with the length of preoperative hospital stay, BMI, ECC time, and PHAI as independent variables. Both multivariable models included a total of 766 patients with complete data available. We chose not to perform data imputation due to the significance of the information for the objectives of our study.

### Ethical Statement

This study followed the principles of the Declaration of Helsinki and was approved by the Research Ethics Committee of Universidade Federal de Santa Maria (Registry number: 29579820.1.0000.5346)^[10]^. The individual patient informed consent was waived. The report of this study was methodically structured in accordance with the guidelines set forth by the Strengthening the Reporting of Observational Studies in Epidemiology (or STROBE) Statement^[11]^.

## RESULTS

A total of 880 patients were included in the study. In the overall sample, the mean age was 61.36 ± 10.744 years, and men comprised 607 (69%) patients. Medical comorbidities include hypertension in 722 (82.3%), DM in 141 (19.4%), and COPD in 42 (4.8%) patients. The most frequent surgical procedure performed was CABG (65.4%), followed by isolated valvular replacement (22.8%) and CABG associated with valvular replacement (8.6%). A total of 275 patients (31.3%) developed postoperative infections. Pulmonary infections (57%) and surgical wound infections (20.2%) were the predominant infection sites. The overall mortality in the complete sample was 85 (9.7%) cases. Table 1 describes the clinical characteristics of the sample and the surgical procedure.

**Table 1:** Additional epidemiological characteristics of the population.

Variables	Available sample size (%)	Mean ± Std^*^ or Total (%)
Age^*^	880 (100)	61.36 ± 10.74
Sex	880 (100)	
Male		607 (69)
Female		273 (31)
SAH	877 (99.70)	722 (82.30)
BMI^*^	793 (90.11)	27.13 ± 4.52
DM	728 (82.70)	
DM (non-insulin-dependent)		101 (13.90)
DM (insulin-dependent)		40 (5.50)
Smoking	851 (96.70)	
Active smoking		179 (21.00)
Former smoker for ≥ 6 months		345 (40.50)
COPD	877 (99.70)	42 (4.80)
Symptoms on admission	879 (99.90)	
No symptoms		66 (4.50)
Stable angina		197 (22.40)
Unstable angina		158 (18.00)
NSTEMI		151 (17.20)
STEMI		72 (8.20)
Heart failure		29 (3.30)
Recent heart attack (< 90 days)	877 (99.70)	131 (14.93)
Ejection fraction^*^	840 (95.45)	58.25 ± 11.79
Carotid disease	527 (59.88)	216 (41.00)
Previous TIA/stroke	507 (57.60)	80 (15.80)
PAD^*^	473 (53.75)	77.28 ± 14.84
Creatinine clearance^*^	864 (98.18)	72.23 ± 27.05
Endocarditis	877 (99.65)	32 (3.60)
Previous atrial fibrillation	877 (99.65)	
Paroxysmal		63 (7.20)
Persistent		01 (0.10)
Permanent		16 (1.80)
Decompensated CHF	876 (99.54)	375 (42.80)
Preoperative INR	643 (73.06)	1.01
Previous cardiac surgery	876 (77.08)	41 (4.70)
Surgical conditions on admission	479 (54.43)	
Elective surgery		212 (44.30)
Urgency surgery		254 (53.00)
Emergency surgery		12 (2.50)
Type of procedure	876 (99.54)	
CABG		573 (65.40)
CABG + VR		75 (8.60)
VR		200 (22.80)
Aortic dissection		15 (1.70)
Aneurysm		04 (0.50)
CABG + aneurysm		03 (0.30)
Other		06 (0.70)

* Mean ± standard deviation

BMI=body mass index; CABG=coronary artery bypass grafting; CHF=congestive heart failure; COPD=chronic obstructive pulmonary disease; DM=diabetes mellitus; INR=international normalized ratio; NSTEMI=non-ST-elevation myocardial infarction; PAD=peripheral arterial disease; SAH=systemic arterial hypertension; STEMI=ST-elevation myocardial infarction; TIA=transient ischemic attack; VR=valve replacement

In the univariate analysis considering the outcome of PHAI, 869 (98.75%) patients met the inclusion criteria. The mean age among patients with PHAI was 1.08 ± 0.05 years higher compared to those without infection (N=869; P<0.001). Compared to patients without PHAI, those with PHAI showed higher occurrences of postoperative stroke by 6.3% (P<0.001), postoperative infarction by 1.3% (P=0.019), reintubation by 14.7% (P<0.001), and reintervention by 13% (P<0.001). The median length of the preoperative hospital stay was 9.44 days among those who developed PHAI, while this number was 8.28 days among those without PHAI (P=0.049). A higher ICU length of stay was registered among patients with PHAI (median of 8.05 days among those with PHAI, compared to 4.35 days; P<0.001). The rate of ICU readmission was 7% in those with PHAI, while this rate was 0.7% in those without it (P<0.001). Patients with PHAI experienced a hospital stay that was 11.81 days longer compared to those without it (28.41 days vs. 16.6 days; P<0.001). The European System for Cardiac Operative Risk Evaluation (EuroSCORE) of patients with postoperative PHAI was 1.5% higher compared to those without it (4.01% vs. 2.51%; P=0.001). Among patients with PHAI, death was observed in 41 individuals (14.9%), indicating a 9.3% higher occurrence compared to the group without PHAI, where this outcome was registered in 33 cases (5.6%), (P<0.001). The other outcomes are shown in Table 2.

**Table 2:** Preoperative characteristics in univariate analysis.

Variables	Sample size (infection)	No infection (N = 594)	Infection (N= 275)	*P*-value	Sample size (death)	No death (N = 795)	Death (N = 85)	*P*-value
Age^*^	869	60.99 ± 10.76	62.07 ± 10.713	0.169	880	61.02 ± 10.648	64.59 ± 11.167	0.004
Sex				0.133				0.001
Male	602	421 (70.9%)	181 (65.8%)		607	562 (70.7%)	45 (52.9%)	
Female	267	173 (29.1%)	94 (34.2%)		273	233 (29.3%)	40 (47.1%)	
SAH	869	484 (81.5%)	231 (84%)	0.366	877	652 (82%)	70 (85.4)	0.448
BMI^*^	787	26.939 ± 4.561	27.579 ± 4.473	0.066	793	27.239 ± 4.537	26.07 ± 4.315	0.046
DM				0.315				0.406
DM (non-insulin- dependent)	101	72 (14.1%)	29 (13.7%)		101	88 (13.3%)	13 (19.1%)	
DM (insulin- dependent)	40	24 (4.7%)	16 (11.7%)		40	36 (5.5%)	4 (5.9%)	
Smoking				0.757				0.862
Active smoking	178 (21.1%)	123 (21.4%)	55 (20.5%)		179 (21%)	163 (21.1%)	16 (20%)	
Former smoker for ≥ 6 months	343 (40.7%)	229 (39.8%)	114 (42.5%)		345 (40.5%)	314 (40.7%)	31 (38.8%)	
COPD	869	22 (3.7%)	20 (7.3%)	0.023	877	32 (4.0%)	10 (12.2%)	0.001
Symptoms on admission				0.067				< 0.001
No symptoms	66 (7.6%)	52 (8.8%)	14 (5.1%)		66 (7.5%)	62 (7.8%)	4 (4.7%)	
Stable angina	197 (22.7%)	140 (23.6%)	57 (20.7%)		197 (22.4%)	190 (23.9%)	7 (8.2%)	
Unstable angina	157 (18.1%)	114 (19.2%)	43 (15.6%)		158 (18.0%)	139 (17.5%)	19 (22.4%)	
NSTEMI	151 (14.7%)	99 (16.7%)	52 (18.9%)		151 (17.2%)	135 (17.0%)	16 (18.8%)	
STEMI	72 (8.3%)	42 (7.1%)	30 (10.9%)		72 (8.2%)	68 (8.6%)	4 (4.7%)	
Heart failure	27 (3.1%)	15 (2.5%)	12 (4.4%)		29 (3.3%)	24 (3.0%)	5 (5.9%)	
Recent heart attack (< 90 days)	131	88 (64.2%)	43 (71.7%)	0.309	131	118 (66.7%)	13 (61.9%)	0.663
Ejection fraction^*^	834	58.99 ± 11.091	56.78 ± 13.002	0.018	840	58.42 ± 11.607	56.65 ± 13.4	0.258
Carotid disease	523	140 (39.3%)	75 (44.9%)	0.226	527	195 (40.2%)	21 (50.0%)	0.216
Previous TIA/stroke	499	40 (11.7%)	38 (24.2%)	< 0.001	507	67 (14.9%)	13 (23.2%)	0.106
PAD^*^	466	76.19 ± 14.049	79.83 ± 16.636	0.016	473	77.05 ± 14.673	79.20 ± 16.274	0.334
Creatinine clearance^*^	858	73.85 ± 26.451	69.13 ± 28.074	0.018	864	74.03 ± 26.622	53.83 ± 24.622	< 0.001
Endocarditis	869	19 (3.2%)	13 (4.7%)	0.266	877	25 (3.1%)	7 (8.5%)	0.013
Previous atrial fibrillation				0.694				< 0.001
Paroxysmal	62 (7.2%)	40 (6.7%)	22 (8.0%)		63 (7.2%)	48 (6.1%)	15 (18.3%)	
Persistent	1 (0.1%)	1 (0.2%)	0		1 (0.1%)	1 (0.1%)	0	
Permanent	15 (1.7%)	9 (10.3%)	6 (2.2%)		16 (1.8%)	12 (1.5%)	4 (4.9%)	
Decompensated CHF	868	244 (41.1%)	126 (45.8%)	0.195	876	329 (41.4%)	46 (56.1%)	0.011
Preoperative INR	639	1.00	1.01	0.269	643	1.01	1.04	0.225
Previous cardiac surgery	869	23 (3.9%)	15 (5.5%)	0.289	876	32 (4.0%)	9 (11.1%)	0.004
Surgical conditions on admission				< 0.001				0.001
Elective surgery	209 (44.6%)	165 (50.3%)	44 (31.2%)		212 (44.3%)	196 (46.1%)	16 (29.6%)	
Urgency surgery	251 (53.5%)	159 (48.5%)	92 (65.2%)		254 (53.0%)	221 (52.0%)	33 (61.1%)	
Emergency surgery	9 (1.9%)	4 (1.2%)	5 (3.5%)		13 (2.7%)	8 (1.9%)	5 (9.3%)	
Type of procedure				0.001				< 0.001
CABG	573 (66.2%)	399 (67.5%)	174 (63.5%)		573 (65.4%)	535 (67.6%)	38 (45.2%)	
CABG + VR	72 (8.3%)	34 (5.8%)	38 (13.9%)		75 (8.6%)	57 (7.2%)	18 (21.4%)	
VR	197 (22.8%)	146 (24.7%)	51 (18.6%)		200 (22.8%)	181 (22.9%)	19 (22.6%)	
Aortic dissection	11 (1.3%)	5 (0.8%)	6 (2.2%)		15 (1.7%)	8 (1.0%)	7 (8.3%)	
Aneurysm	4 (0.5%)	3 (0.5%)	1 (0.4%)		4 (0.5%)	4 (0.5%)	0	
CABG + aneurysm	3 (0.3%)	2 (0.3%)	1 (0.4%)		3 (0.3%)	2 (0.3%)	1 (1.2%)	
Other	5 (0.6%)	2 (0.3%)	3 (1.1%)		6 (0.7%)	5 (0.6%)	1 (1.2%)	

* Mean ± standard deviation

BMI=body mass index; CABG=coronary artery bypass grafting; CHF=congestive heart failure; COPD=chronic obstructive pulmonary disease; DM=diabetes mellitus; INR=international normalized ratio; NSTEMI=non-ST-elevation myocardial infarction; PAD=peripheral arterial disease; SAH=systemic arterial hypertension; STEMI=ST-elevation myocardial infarction; TIA=transient ischemic attack; VR=valve replacement

For the death outcome in the univariate analysis, an average difference of 3.57 years was observed among patients who died before hospital discharge, compared to those who did not (64.59 ± 11.167 years vs. 61.02 ± 10.648 years; P=0.004). In patients who died before hospital discharge, there was a 7.5% higher incidence of postoperative stroke (10.5% vs. 3.0%; P=0.001), a 50.3% higher likelihood of reintubation (52.8% vs. 2.5%; P<0.001), a 6.4% greater need for surgical reintervention (11.6% vs. 5.2%; P=0.002), a 13.3% higher rate of ICU readmission (15.2% vs. 1.9%; P<0.001), and a 2.62% increase in EuroSCORE compared to those who did not die (5.32% vs. 2.70%; P<0.001). Table 3 displays the additional results.

**Table 3:** Postoperative outcomes in univariate analysis.

Variables	Sample size (infection)	No infection (N= 594)	Infection (N= 275)	*P*-value	Sample size (death)	No death (N= 795)	Death (N= 85)	*P*-value
ECC time^*^	846	100.84 ± 32.450	110.49 ± 39.425	0.001	850	101.91 ± 33.328	129.28 ± 50.108	< 0.001
CPB time^*^	845	74.39 ± 26.388	80.28 ± 31.361	0.008	850	74.88 ± 27.050	92.30 ± 35.592	< 0.001
Shock				< 0.001				0.007
Cardiogenic shock	40 (54.8%)	23 (85.2%)	17 (37.0%)		46 (58.2%)	29 (76.3%)	17 (41.5%)	
Septic shock	16 (21.9%)	0	16 (34.8%)		16 (20.3%)	4 (10.5%)	12 (29.3%)	
Mixed shock	17 (23.3%)	4 (14.8%)	13 (28.3%)		17 (21.5%)	5 (13.2%)	12 (29.3%)	
CRP	868	19 (3.2%)	39 (14.2%)	< 0.001	871	20 (2.5%)	41 (53.9%)	< 0.001
Arrhythmia (as of ECC exit)	569	272 (69.4%)	141 (79.7%)	0.007	571	375 (71.8%)	39 (79.6%)	0.245
Postoperative stroke	869	10 (1.7%)	22 (8.0%)	< 0.001	871	24 (3.0%)	8 (10.5%)	0.001
Postoperative infarction	824	1 (0.2%)	4 (1.5%)	0.019	830	4 (0.5%)	1 (1.4%)	0.383
Reintubation	847	6 (1.0%)	42 (15.7%)	< 0.001	848	20 (2.5%)	28 (52.8%)	< 0.001
Reintervention				< 0.001				0.002
Surgical reintervention	465	7 (2.1%)	20 (14.4%)		466	22 (5.2%)	5 (11.6%)	
Hemodynamic reintervention		0	1 (0.7%)			0	1 (2.3%)	
Surgical reintervention, causes				0.638				0.638
Mediastinitis	2	0	2 (9.1%)		2 (6.9%)	1 (4.5%)	1 (14.3%)	
Increased bleeding	8	4 (57.1%)	4 (18.2%)		8 (27.6%)	6 (27.3%)	2 (28.6%)	
Endocarditis	0	0	0		0	0	0	
Cardiac tamponade	3	0	3 (16.3%)		3 (10.3%)	3 (13.6%)	0	
Other	16	3 (42.9%)	13 (59.1%)		16 (55.2%)	12 (54.5%)	4 (57.1%)	
ICU stay, median (IQR)	866	4.35	8.05	< 0.001	877	5.37	6.45	0.381
ICU readmission	832	4 (0.7%)	18 (7%)	< 0.001	840	15 (1.9%)	7 (15.2%)	< 0.001
Tracheostomy	633	0	9 (4.3%)	< 0.001	664	7 (1.1%)	2 (4.3%)	0.074
Preoperative hospital stay, median (IQR)	869	8.28	9.44	0.049	880	8.50	9.76	0.170
Total hospital stay, median (IQR)	869	16.6	28.41	< 0.001	880	20.14	20.65	0.836
EuroSCORE, median (IQR)	428	2.51%	4.01%	0.001	433	2.70%	5.32%	< 0.001

* Mean ± standard deviation

CPB=cardiopulmonary bypass; CRP=cardiorespiratory arrest; ECC=extracorporeal circulation; EuroSCORE=European System for Cardiac Operative Risk Evaluation; ICU=intensive care unit; IQR=interquartile range

After binary logistic regression, a longer preoperative hospital stay predicted higher odds of PHAI (OR 1.024; 95% CI 1.005-1.044; P=0.009), as shown in the analysis presented in Table 4. This suggests an estimate of 2.4% higher odds for PHAI for each additional day. Each additional increment of one unit per kg/m^2^ increased the odds of PHAI by 4.3% (OR 1.043; 95% CI 1.008-1.079; P=0.015). The odds of PHAI were also increased by 0.7% for each additional minute in ECC time (OR 1.007; 95% CI 1.003-1.012; P<0.001).

**Table 4:** Predictive factors for postoperative hospital-acquired infection in multivariate analysis.

Variable N= 766	Odds ratio	95% CI	Significance
Preoperative hospital stay (days)	1.024	1.005 - 1.044	0.009
BMI (kg/m^2^)	1.043	1.008 - 1.079	0.015
ECC time (minutes)	1.007	1.003 - 1.012	<0.001

BMI=body mass index; CI=confidence interval; ECC=extracorporeal circulation

Similarly, in the analysis presented in Table 5, the longer ECC time was also associated with a 1.2% higher odds of death outcome (OR 1.012; 95% CI 1.006-1.019; P<0.001). PHAI increased the odds of mortality by 2.418 times (OR 2.418; 95% CI 1.385-4.233; P=0.001).

**Table 5:** Predicted factors for in-hospital mortality in multivariate analysis.

Variable N= 766	Odds ratio	95% CI	Significance
Length of preoperative hospital stay (days)	0.052	0.008 - 0.347	0.233
BMI (kg/m^2^)	0.944	0.887 - 1.003	0.067
ECC time (minutes)	1.012	1.006 - 1.019	<0.001
PHAI	2.418	1.385 - 4.233	0.001

BMI=body mass index; CI=confidence interval; ECC=extracorporeal circulation; PHAI=postoperative hospital-acquired infection

## DISCUSSION

PHAI has been recognized as a key factor of morbidity and mortality, which was reaffirmed by the current study that demonstrated that PHAI is an independent factor for mortality, alongside extended ECC time, which also operates as an independent risk factor associated with death following cardiac surgery^[12,13]^. We also demonstrated that BMI, ECC time, and prolonged preoperative hospital stay are independent factors related to PHAI after cardiac surgery. The incidence of PHAI following cardiac surgery in our study reached 31.6%, exceeding the range of 0.5% to 21% commonly observed in previous studies^[4,14-16]^. These distinctions may be accounted for by a range of factors, such as variations in the methodology or diagnostic criteria utilized, the exclusion of patients with prior infections, the specific infection control strategies adopted at individual centers, and the complexity of the surgical procedures undertaken. Similarly with previous studies, pulmonary (57%) and surgical wound (20.2%) infections were the most common sites of infection^[4,14,17,18]^. Additionally, the overall mortality rate in our study was 9.7%, which exceeded the typical range of 3.14% to 4.7% observed in previous studies^[3,19]^.

In line with earlier studies, our study underscores the relationship between body weight and PHAI risk^[7,15]^. Patients with higher BMI exhibited a 1.043-fold greater likelihood of PHAI (OR 1.043; 95% CI 1.008-1.079; P=0.015), which can be attributed to the impact of obesity on treatment outcomes. These challenges include difficulties in maintaining tissue sterility, achieving adequate serum levels of prophylactic antibiotics, and addressing compromised perfusion in adipose tissue^[4]^. We also found that BMI was not established as an independent risk factor for mortality following cardiac surgery, a consistent trend with prior literature^[20]^.

Prolonged ECC time increased the risk of PHAI following cardiac surgery by a factor of 1.007 in our study (OR 1.007; 95% CI 1.003-1.012; P<0.001). Kilic et al. demonstrated a 1.71-fold rise in pneumonia odds among patients undergoing CPB for over 100 minutes^[21]^. Additionally, Allou et al.^[6]^ reported a 2.98-fold higher risk of postoperative pneumonia when CPB time surpassed 60 minutes. These combined results underscore the conclusion that prolonged surgical procedures, whether characterized by CPB or ECC time, amplify the risk of PHAI. Previous research has established that individuals subjected to ECC face an elevated susceptibility to colonization and subsequent infection with multidrug-resistant bacteria^[2,22]^. Furthermore, these infections have been linked to heightened mortality rates. Prolonged ECC time reflects the complexity of cardiac surgery and increases the risk of hemorrhages, and we found that ECC time was an independent factor for mortality^[23]^.

We showed that an extended length of hospital stay prior to cardiac surgery was associated with a 1.024-fold increased risk of PHAI complications (OR 1.024; 95% CI 1.005-1.044; P=0.009). The association between prolonged preoperative hospital stay and PHAI was previously reported in noncardiac surgical context, and authors suggest that the illness severity and exposure to hospital's microorganisms might be causes for increased infection rates^[7,16,21]^. The American College of Surgeons National Surgical Quality Improvement Project (or ACS NSQIP) revealed 21% higher incidence of SSI, pneumonia, and urinary tract infections among patients who were hospitalized preoperatively^[9]^. The researchers furthermore underscored colonization by healthcare-associated bacteria and the presence of catheters as potential risk factors in this regard^[9]^. This prolonged hospitalization contributes to escalated costs, a cornerstone issue for the Brazilian public health system, an 190 million people coverage health system^[1]^. A Brazilian study focusing on costs of the public health system showed that patients with PHAIs experienced a reimbursement cost per hospitalization 75% higher than patients without PHAIs, amounting to US$ 2721 vs. US$ 1553^[1]^.

In our study, we observed that PHAIs following cardiac surgery increase the risk of mortality by 2.418-fold (OR 2.418; 95% CI 1.385-4.233; P=0.001). Our findings are aligned with previous studies, such as the prospective observational study conducted by Varga-Martínez et al., which involved 1097 adult patients and revealed that nosocomial infection stood as the primary risk factor for mortality in patients undergoing cardiac surgery (OR 6.23, 95% CI 2.49-15.63, P< 0.001)^[4]^. Another investigation by Lola et al. found that the mortality rate was 25% among patients with infections and 3.48% among all patients who underwent cardiac surgery, in contrast to the 5.4% mortality rate among patients who did not develop early postoperative infections after cardiac surgery^[3]^.

### Limitations

Our study has certain limitations. Firstly, being a single-center study, the findings and conclusions may not be fully generalizable to the broader population of Brazil or other countries facing comparable circumstances. Variations in healthcare practices, patient demographics, and regional factors might influence outcomes differently in multicentric or diverse settings. Secondly, the retrospective nature of our study introduces inherent biases associated with the review of medical records.

## CONCLUSION

We demonstrated that extended preoperative hospital stay, BMI, and ECC time are independent risk factors for the development of PHAI. Additionally, both PHAI incidence and ECC time predict mortality after cardiac surgery.
