# ERRATUM

**DOI:** 10.21470/1678-9741-2024-0111e

**Published:** 2025-04-03

In the article “Predictors of Postoperative Hospital-Acquired Infection and Mortality Following Cardiac Surgery in a Low-Income Country: A Retrospective Cohort Study”, DOI https://doi.org/10.21470/1678-9741-2024-0111, published in the Brazilian Journal of Cardiovascular Surgery, 40.2, the corresponding author’s email address was incorrect.

In the published article, the information was:

Correspondence Address:

Camila Sales Fagundes

https://orcid.org/0000-0002-6895-0702

Congonhas Street, Nº 140, Santa Maria, RS, Brazil

Zip Code: 97105-050

E-mail: camila@sbccv.org.br

The correct information is:

Correspondence Address:

Camila Sales Fagundes

https://orcid.org/0000-0002-6895-0702

Congonhas Street, Nº 140, Santa Maria, RS, Brazil

Zip Code: 97105-050

E-mail: camila.fafa3@gmail.com

BJCVS apologizes for this error that has now been corrected.

Accepted on April 2^nd^, 2025.

